# Strategies in asymmetric catalysis

**DOI:** 10.3762/bjoc.13.8

**Published:** 2017-01-10

**Authors:** Tehshik P Yoon

**Affiliations:** 1 Department of Chemistry, University of Wisconsin–Madison, 1101 University Avenue, Madison, WI 53706, USA

**Keywords:** asymmetric catalysis, enantioselectivity, stereoselectivity

The stereochemistry of an organic compound can have a profound influence on many of its most important properties. For example, the enantiomeric forms of a drug molecule can have completely different biological effects, and polymers that differ only in the stereochemistry of their backbones can have quite dissimilar macroscopic physical characteristics. Control over the stereochemical outcome of organic reactions has thus long been recognized as a central concern across multiple sectors of modern synthetic chemistry.

In 2001, the Nobel Prize in Chemistry was awarded to Knowles, Sharpless, and Noyori for their work on the use of chiral catalysts for highly enantioselective reactions. Those of us with research interests in stereoselective synthesis lauded this award because it celebrated the creative insights of these pioneers in asymmetric catalysis and because it marked a general recognition that enantioselective catalysis has had a significant practical impact on the broader field of organic synthesis.

Despite the remarkable progress in this field over the years, however, the rational design and discovery of enantioselective reactions remains a challenging endeavor. Part of the difficulty is intrinsic: subtle energy differences of only a few kilocalories per mole can nevertheless result in very high enantioselectivities in a given reaction. These effects can be hard to predict, to rationalize, and to generalize to diverse reaction types. Moreover, even small deviations from the optimal catalyst and substrate structures can sometimes result in poor results.

Thus, the design of asymmetric catalytic systems remains an active and vital research area. Current progress in the field of asymmetric catalysis continues to be driven both by conceptual innovations as well as demonstrations of the applicability of enantioselective catalytic reactions to increasingly complex synthetic problems.

The articles collected in this Thematic Series for the *Beilstein Journal of Organic Chemistry* provide a snapshot of both types of efforts. Several of these papers report new catalyst designs or relatively new concepts in catalytic stereocontrol. Others document the application of catalytic asymmetric methods to the streamlined synthesis of complex and structurally unusual organic molecules. I would like to express my gratitude to the authors and researchers who have contributed articles to this Thematic Series. The creativity reflected in this set of papers augurs well for the continued vitality of what I believe is an important research endeavor. I hope you will agree.

Madison, December 2016

Tehshik P. Yoon

